# Post-laser in situ keratomileusis (LASIK) epithelial ingrowth after interface fluid syndrome

**DOI:** 10.3205/oc000267

**Published:** 2026-02-17

**Authors:** Emiel Matthys, Isabelle Saelens, Heleen Delbeke

**Affiliations:** 1 Department of Ophthalmology, University Hospitals Leuven, Belgium

**Keywords:** LASIK, interface fluid syndrome, epithelial ingrowth

## Abstract

**Objectives:**

We present a case of epithelial ingrowth after late onset interface fluid syndrome, a rare complication after laser in situ keratomileusis (LASIK).

**Methods:**

This patient presented herself to the emergency department with corneal oedema due to endothelial failure after multiple intraocular surgeries. Anterior segment optical coherence tomography (OCT) showed a fluid filled pocket in the LASIK interface, 20 years after LASIK surgery.

**Results:**

As a result, she was diagnosed with late onset interface fluid syndrome which resolved after endothelial transplantation (DMEK). However, 3 months after surgery, epithelial ingrowth in the LASIK interface was noticed. We adopted a conservative approach as there was no progression after 3 weeks.

**Conclusions:**

Epithelial ingrowth is a rare complication after DMEK, which might be caused by interface fluid induced flap lifting and further facilitated by intraoperative flap manipulation during graft unfolding.

## Introduction

Laser in situ keratomileusis (LASIK) is the most widely performed laser refractive surgery technique to date with excellent long-term effectiveness, predictability and safety [1]. However, the creation of an interface between the flap and the corneal stroma creates the potential for flap related complications like flap dislocation, infectious keratitis, diffuse lamellar keratitis, interface fluid syndrome and post-LASIK epithelial ingrowth [2], [3], [4], [5]. We present a case of epithelial ingrowth after late onset interface fluid syndrome, a rare complication after LASIK. 

## Case description

**Figure 1:**
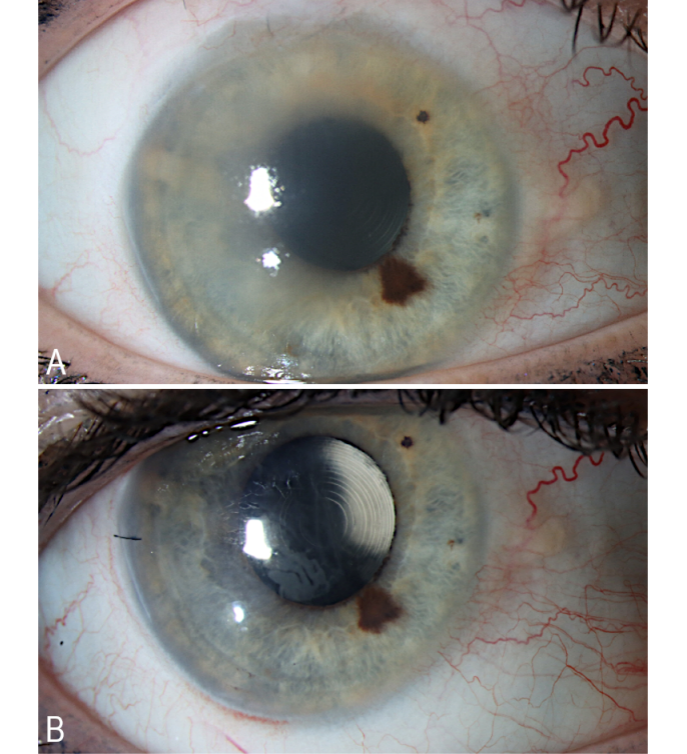
Anterior segment imaging. A: Corneal oedema pre-DMEK. B: Post-LASIK epithelial ingrowth after DMEK – in the area of previous corneal oedema

**Figure 2:**
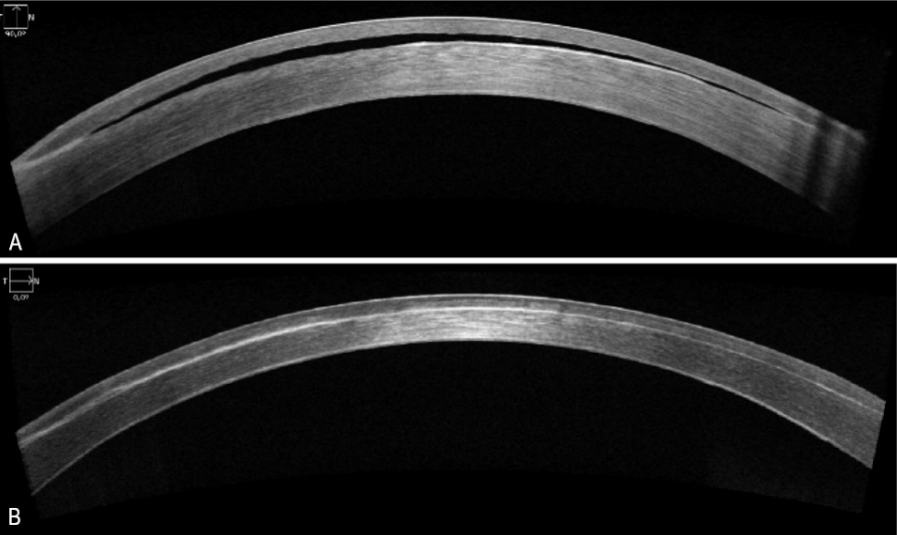
Corneal OCT. A: Corneal OCT showing interface fluid. B: Corneal OCT showing resolution of interface fluid after DMEK surgery

A 55-years-old female patient presented herself to the emergency department due to blurred vision with acute onset. She had a history of high myopic refractive error (–16D) with bilateral Implantable Collamer Lens (ICL) implantation and laser-assisted in situ keratomileusis (LASIK), with a redo LASIK at the right eye 4 years after the initial LASIK procedure. She had received cataract surgery with multifocal intra-ocular lens implantation 15 years after her primary surgery. Furthermore, she suffered from advanced normal tension glaucoma with previous Microshunt implantation in the right eye. Upon presentation at the emergency department, she had a decrease in best corrected visual acuity (BCVA) at her right eye; Snellen 0.16 compared to Snellen 0.3 at her last visit 3 weeks before. Intra-ocular pressure, measured by Goldmann applanation tonometry, was 11 mmHg in the right and 10 mmHg in the left eye with a target pressure of 10 mmHg. Slit lamp examination revealed diffuse corneal edema (Figure 1A (Fig. 1)) with normal symmetrical corneal sensitivity. Clinical exam was otherwise unremarkable. Conjunctival swab to rule out herpetic keratitis was negative and topical treatment consisting of sodium chloride 5% eyedrops and preservative free Dexamethasone 0.1% eyedrops were initiated. During a follow-up consultation at our corneal clinic, specular microscopy of the corneal endothelium showed marked endothelial loss with optical coherence tomography (OCT) of the cornea showing an interface fluid pocket (Figure 2A (Fig. 2)). As a result, stage 3 interface fluid syndrome was diagnosed. 

**Figure 3:**
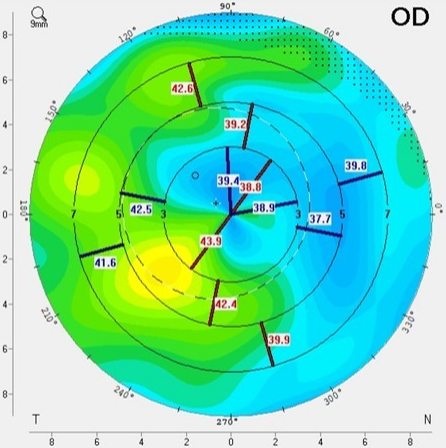
Irregular astigmatism on Scheimpflug imaging. Scheimpflug imaging with Pentacam^®^ showing irregular astigmatism in the central 5 mm corneal zone due to epithelial ingrowth in the LASIK interface

Uneventful Descemet membrane endothelial keratoplasty (DMEK) surgery was performed with a marked postoperative increase in visual acuity (Snellen 0.5, 3 months postoperative). OCT of the cornea 2 months after surgery showed complete resolution of the interface fluid pocket with complete attachment of the Descemet membrane (Figure 2B (Fig. 2)). Three months after surgery, epithelial ingrowth in the interface was noticed (Figure 1B (Fig. 1)). This epithelial ingrowth did cause irregular astigmatism observed with Scheimpflug imaging (Figure 3 (Fig. 3)). However, as her visual acuity was significantly improved, the patient preferred a watchful waiting approach as she had already undergone a myriad of eye surgeries in the past few years.

## Discussion

Interface fluid syndrome is an uncommon, but vision-threatening flap-related complication occurring 1 week to a few months after uneventful LASIK surgery. Interface fluid develops as a result of endothelial cell dysfunction. The most common aetiology is a steroid-induced rise in intraocular pressure with pressure-induced endothelial failure [4], [6]. When interface fluid is suspected, the intraocular pressure should be measured with applanation tonometry at the peripheral cornea, as the interface fluid may have a cushioning effect with consequently falsely low IOP measurements [4], [6], [7].

When interface fluid develops without a raise in intraocular pressure, the differential diagnosis includes other causes of endothelial failure such as Fuchs dystrophy or pseudophakic bullous keratopathy [4], [6].

The interface fluid-pocket can be visualised by corneal OCT or even slit lamp examination in more pronounced cases [2], [4], [6], [8]. By correlating the results of confocal microscopy, histopathology and ultrastructural findings, Dawson and colleagues developed a staging system:

- Stage 1: Mild to moderate swelling of the LASIK wound with no to minimal haze
- Stage 2: Moderate to severe swelling of the LASIK wound with a diffuse, smudgy nongranular interface haze and build-up of small fluid pockets
- Stage 3: A large, diffuse interface fluid pocket with a surrounding haze [4]

The patient in this case presented with interface fluid syndrome, stage 3. 

In the event of visual decline after LASIK, one must make the differential diagnosis with diffuse lamellar keratitis (DLK) [4]. DLK is a non-specific inflammatory condition occurring 1–5 days after LASIK surgery. This is in contrast with interface fluid syndrome, which occurs at least one week after LASIK surgery [3]. White blood cells (WBC) infiltrate into the corneal stroma and the LASIK interface resulting in a granular appearance. Therefore, it can be easily mistaken for mild interface fluid syndrome, which appears as a more smudgy, non-granular haze [2]. Patients often present with a painful red eye within the first week after LASIK surgery. Linebarger and colleagues describe 4 stages (*Stage 1*: peripheral WBC infiltration – to – *Stage 4*: dense haze with scarring and flap necrosis) [9]. DLK is responsive to aggressive topical steroids in mild stages, combined with oral steroids in advanced stages. Flap lifting is controversial due to the possibility of tissue loss [3].

Post-LASIK epithelial ingrowth is a rare complication following LASIK surgery that is characterised by the ingrowth of corneal epithelium cells at the interface [3]. Reported incidences vary from 0–3.9% in primary treatment cases and 10–20% in retreatment cases [5]. There is a wide spectrum of clinical presentations, from mostly asymptomatic (64%) to visual disturbance (decrease in visual acuity, glare) or even flap melting [5]. There is a dual pathogenesis of interface epithelial ingrowth. During flap creation there is a possibility of epithelial cell implantation, resulting in nests of epithelial cells with minimal proliferative ability. Another possibility is migration of epithelial cells with proliferative capacity from the flap edge due to flap dislocation or poor flap adhesion [5], [10]. Flap lifting in retreatment, trauma or after other forms of ocular surgery facilitates the migration of epithelial cells as well. Proliferation can lead to fistula formation, resulting in extensive proliferative post-LASIK epithelial ingrowth [5]. 

The Probst/Machat classification describes a clinical spectrum of 4 grades, requiring no to urgent treatment.

- Grade 1: Thin, non-progressive growth within 2 mm of the flap edge; requiring no treatment
- Grade 2: Thicker, slowly progressive growth within 2 mm of the flap edge which is often rolled or grey; requiring non-urgent treatment within 2–3 weeks
- Grade 3: Pronounced, progressive, opaque growth with areas of necrotic cells creating a peripheral confluent haze; requiring urgent treatment
- Grade 4: Aggressive growth of strands of epithelial cells invading towards the visual axis; requiring urgent treatment [5]

Modifiable risk factors for post-LASIK epithelial ingrowth are flap dislocation, flap lifting and corneal epithelial injury, with subsequent hydration causing flap lifting, besides the applied surgical technique [5], [10]. In our case there are 3 possible hypotheses for the aetiology of epithelial ingrowth. The first hypothesis involves the migration of epithelial cells due to poor flap adhesion, caused by the interface fluid (Figure 1B (Fig. 1), epithelial cells observed in the area of previous corneal oedema) combined with possible intraoperative flap manipulation during DMEK surgery (e.g. corneal tapping during graft unfolding). This is in line with the presumed pathophysiology of Luiz et al., in which they hypothesised that the interface fluid lifts the flap edge, creating a route for epithelial ingrowth [8]. 

The second hypothesis is based on the in vitro investigations of Davanger and Olsen on epithelial-endothelial interaction trough a corneal perforation (i.c. sclero-corneal incision) [11]. Their experiments demonstrated that if the endothelial quality is reduced, there is a high tendency of epithelial ingrowth trough corneal perforations. This due to the loss of normal contact inhibition by the endothelial cells [11]. Before DMEK surgery, there was a reduced quality in endothelial function. After the surgery, there remains a peripheral zone of pathologic endothelium which may facilitate epithelial ingrowth postoperatively. However, this theory applies more to epithelial intra-ocular ingrowth and less to epithelial ingrowth in the LASIK interface.

The third and final hypothesis involves the possibility of longstanding nests of epithelial cells in the interface, which might have been masked due to the corneal edema. These nests may have been there for years as they are presumed to have a low proliferative capacity. Our patient has normal-tension glaucoma and underwent multiple ocular procedures for which she had numerous follow-ups after her LASIK surgery. There were no previous observations of epithelial ingrowth, making this hypothesis the least plausible.

The patient showed subtle zones of epithelial nests in the flap interface. These nests are presumed to have a low proliferative capacity [5]. Furthermore, the patient was asymptomatic, and no fistula was detected. Therefore, we opted for a conservative approach. Follow-up consultation 3 weeks later, showed no signs of proliferative capacity.

## Conclusion

Interface fluid syndrome can be caused by non-pressure related causes of endothelial failure, years after LASIK surgery. We describe a rare case of epithelial ingrowth after late onset interface fluid syndrome. Epithelial ingrowth might be caused by interface fluid induced flap lifting and further facilitated by intraoperative flap manipulation during graft unfolding in DMEK surgery.

## Notes

### Acknowledgements

H. Delbeke is the patient’s treating ophthalmologist. She provided the data for this case report. 

I. Saelens provided useful insights in the underlying aetiology of epithelial ingrowth after interface fluid syndrome. 

E. Matthys put in the effort to write the case.

### Competing interests

The authors declare that they have no competing interests.
